# Socioeconomic gradients in early childhood health: evidence from Bangladesh and Nepal

**DOI:** 10.1186/s12939-016-0364-2

**Published:** 2016-05-16

**Authors:** Satis Devkota, Bibhudutta Panda

**Affiliations:** University of Minnesota-Morris, Division of Social Science, 600 East 4th Street, Morris, 56267 MN USA

**Keywords:** Socioeconomic gradients, Early childhood health, Health disparity, Bangladesh, Nepal

## Abstract

**Background:**

A large literature has developed researching the origins of socioeconomic gradients in child health in developed countries. Particularly, this research examines the age at which these gradient effects emerge and how they change across different stages of childhood. However, similar research on developing countries is limited.

**Methods:**

This paper examines the socioeconomic gradients in early childhood health in two developing countries, Bangladesh and Nepal using the 2011 Demographic and Health Surveys. The paper separately studies two measures of household socioeconomic status: household wealth and maternal educational attainment. Two anthropometric measures of early childhood health, height-for-age and weight-for-age Z scores for 0–59 months of children, are used for our empirical exercise. The paper uses both non-parametric and multivariate ordinary least squares approaches to examine at what age socioeconomic disparities in health emerge, and investigates if these disparities increase with age in early childhood.

**Results:**

The paper provides significant evidence of age-specific socioeconomic gradients in early childhood health in both countries. Health disparities in household wealth exist in both countries. This disparity emerges in the first 11 months of life, and is particularly severe for children from the poorest quintile. On the other hand, while the emergence of maternal education gradients during the first 11 months is sensitive to the choice of childhood health measure, the study finds the children of mothers with higher education to enjoy significantly higher health outcomes in comparison to those with lower education. However, controlling for father’s education weakens the effects of maternal education on child health in both countries. Further, the paper does not find statistically significant evidence where socioeconomic gradients in health increase with age in early childhood.

**Conclusions:**

Our study concludes that socioeconomic disparities in health outcomes exist even in very early childhood in Bangladesh and Nepal. This has important implications for targeted policy interventions in the form of food security and nutrition supplement programs, free provision of health care, and maternal education in both countries.

**Electronic supplementary material:**

The online version of this article (doi:10.1186/s12939-016-0364-2) contains supplementary material, which is available to authorized users.

## Background

Socioeconomic disparities in child health have been central to public health research [1–3]. The effect of household socioeconomic status on child health, also known as a gradient, operates through various channels. Children from poorer socioeconomic status may lack access to adequate nutritional food, maternal and child care practices, and may face other environmental risk conditions, leading to poor health outcomes [4].

A large literature has evolved researching the origins of socioeconomic gradients in child health, particularly, examining the age at which these gradient effects emerge and how they change across different stages of childhood. In two seminal studies, Case et al. [1] and Currie and Stabile [5] found that the effect of household income on child health accumulates with age in the U.S. and Canada, respectively. In other words, this effect is stronger among older children. This implies that gradients observed in adult life have antecedents in early childhood. Case et al. [1] argue that children from poorer socioeconomic status are not only more vulnerable to chronic conditions, they are also less likely to effectively manage these conditions through appropriate health care in the long run, causing the health disparity between children from poorer and richer households to increase with age. Currie and Stabile [5], meanwhile, found little variations in the long run effect of health shocks (chronic conditions) among children from different socioeconomic groups. They attributed the steepening gradient effect to the greater likelihood of health shocks on children from lower socioeconomic strata.

The subsequent debate on this view has largely remained elusive. For example, separate studies from the U.S. [6], U.K. [2, 7, 8], Australia [9] and Germany [10] while documenting significant association between socioeconomic status and child health, find little or no evidence of this association getting stronger with age. Further research to contribute this debate will certainly be a valued addition.

Socioeconomic gradients in child health are expected to be more pronounced in developing countries due to widespread disparities in socioeconomic status. Nevertheless, the literature that examines such gradient effects includes only a handful of studies [3, 11–15], and is rather divided if socioeconomic gradients in health increase with child age. This paper aims to contribute to this on-going debate in three important ways. First, we examine socioeconomic gradients in child health in two low-income economies, Bangladesh and Nepal. Bangladesh has experienced significant economic and educational gains over the last two decades, and yet 43.3 % of its population still lives below the absolute poverty line of $1.25 per day [16]. Similarly, only 28.8 % of women have completed secondary schooling [17]. On the other hand, Nepal is still recovering from a decade long civil war and political instability. In this context, 23.7 % of Nepalese population lives below the absolute poverty line [16] and only 13.7 % of women have completed secondary schooling in Nepal [17]. In light of these glaring socioeconomic inequalities in both countries, identifying socioeconomic gradients in child health has significant implications for their health policies and long-run human capital outcomes.

Second, our study focuses on socioeconomic disparities in health in early childhood (children aged, 0–59 months) because early childhood health outcomes have implications for cognitive development and school achievement [18, 19], and for long-run adult outcomes pertaining to health, education, and labor market performance [20]. Further, a recent paper documents significant socioeconomic gradients in health noting the stronger effects with age among very young children of 0–23 months from four developing countries [3].

Finally, our study primarily focuses on objective measures of health in contrast to the widely used parents-assessed subjective measures that characterize this literature. Subjective measures of health can be influenced by socioeconomic characteristics of the household, and emotional and psychological conditions of the respondent [21], and can potentially bias the observed gradient effects. Further, Cameron and Williams [12] argue that the prevalence of acute conditions, such as diarrhea and fever, forms the basis of parent-assessed health in developing countries unlike the chronic conditions in developed countries. While chronic conditions are more likely to emerge as children age, acute conditions are less prevalent among older children, leading to a lack of stronger gradient effect among older children in developing countries. Our emphasis on two widely used objective measures, Height-for-Age Z score (HAZ) and Weight-for-Age Z score (WAZ), helps to isolate such biases [22].

Guided by the above discussion, we reiterate that our research primarily examines socioeconomic gradients in early childhood health in Bangladesh and Nepal. This study systematically examines the age at which socioeconomic disparities in health emerge and investigates if these disparities in health increase with age in early childhood.

## Methods

### Data

We use the 2011 Demographic and Health Surveys (DHS) data for both Bangladesh and Nepal. The Bangladesh DHS was conducted by the Ministry of Health and Family Welfare, Bangladesh, and the Nepal DHS was conducted by the Ministry of Health and Population, Nepal, in collaboration with the U.S. Agency for International Development (USAID). Both these surveys provide rich information on child and maternal health conditions, demographic and socioeconomic characteristics at individual and household level.

Both these surveys are two-stage stratified nationally representative random samples. For sampling purposes, Bangladesh was stratified into 20 strata covering seven divisions of the country. Samples were selected independently from these strata in two stages. In the first stage of the survey, 600 Primary Sampling Units (PSUs) (207 in urban areas and 393 in rural areas) were randomly selected with probability proportional to size. A sample of 30 households on average per PSU was selected in the second stage of the survey. The Bangladesh DHS resulted from completed interviews of 17,842 eligible women aged 12–49 years (98 % response rate) and from 17,141 selected households (98 % response rate). Similarly, for sampling purposes, Nepal was divided into 25 sampling strata covering the three ecological and five developmental regions of the country. Samples were selected randomly from each stratum in two stages. The first stage of the Nepal survey selected 289 PSUs (95 in urban areas and 194 in rural areas) using a probability proportional to size strategy. The second stage randomly selected 35 households per urban PSU and 40 households per rural PSU. The final survey resulted from successful interviews of 12,674 eligible women aged 15–49 years (98 % response rate) and from 10,826 households (99 % response rate). Further details on sample design can be found in the DHS final reports for these surveys [23, 24].

**Early childhood health measures:** This study uses DHS provided HAZ and WAZ scores of children up to 59 months as the measures of early childhood health. Both Bangladesh and Nepal DHS collected anthropometric data by measuring the height and weight for all children up to 59 months in the selected households. The DHS converted these height and weight measures to their corresponding Z-scores (i.e. HAZ and WAZ scores, respectively) using the World Health Organization (WHO) Child Growth Standards reference median. These growth standards are based on an international sample of diverse group of 8,440 children from six countries (Brazil, Ghana, India, Norway, Oman, and the United States) living under optimum genetic growth conditions [22]. These two standardized Z-scores are computed as the numbers of standard deviation away from WHO Child Growth Standards reference median [22]. The HAZ scores reflect long-term linear growth, and capture the effects of chronic malnutrition. On the other hand, the WAZ score is a composite score reflecting both chronic as well as acute malnutrition. Children with HAZ scores 2-SD below the WHO reference median are considered to be stunted where as children with WAZ scores 2-SD below the corresponding reference median are considered to be underweight [23, 24]. The current study is based on the weighted sample of 7683 children from Bangladesh and 2380 children from Nepal with valid information on anthropometric measurements (height and weight) and age who were usual members of the selected households.

**Socioeconomic status measures:** We use household economic status and maternal education as the measures of household socioeconomic status. Because of the lack of data on household income and consumption expenditure in these surveys, we use the DHS wealth index as a proxy for household economic status. DHS constructs this index using the information on household assets and household access to various utility services, and divides the population into five equal quintiles [25]. Similarly, we divide the maternal educational attainment into three categories: mothers with no education, mothers who have attended primary education (i.e. up to five years of schooling), and attended secondary or higher level education (henceforth, secondary education)(i.e. more than five years of schooling).

**Control variables:** We control for various confounding factors by controlling for child specific factors such as child’s age (by introducing dummies for 0–11, 12–35 and 36–59 months age groups), child’s gender, birth size and birth order, mother specific factors such as mother’s age, nutritional status (height/Body Mass Index (BMI)), father specific factors such as father’s age and education, and household specific factors such as religion, rural/urban household and household size. We also control for maternal education while examining gradients in household wealth, and household wealth status while examining maternal education gradients. To control for maternal nutritional status, we use mother’s height as a control variable while analyzing childhood HAZ scores, and use mother’s BMI while analyzing childhood WAZ scores. In addition, we also control for regional heterogeneity in child health by using regional dummies for seven major divisions in Bangladesh, and for five developmental regions in Nepal.

The final sample size (weighted) for Bangladesh is 7505, and 2342 for Nepal for which we have complete information on all the required variables.^1^ This means that we are loosing 2.32 % and 1.6 % of the sample for Bangladesh and Nepal, respectively due to missing observations in control variables. However, we found that the data are missing completely at random, hence should not bias our estimates from statistical analysis. Figure S1 in the Additional file 1 demonstrates the sample selection procedure for both countries.

### Statistical analysis

We conduct the statistical analysis in two phases. Following Fernald et al. [3], first we use a graphical analysis, which is non-parametric in nature. To visually examine if household wealth gradient in child health increases with age, we plot the smoothed values of health outcomes (HAZ/WAZ scores) as a function of children’s age for the richest quintile against the combined lower four quintiles using a Kernel-weighted local polynomial smoothing. In other words, we plot a smoothed curve that represents the mean values of HAZ/WAZ scores at each age (in months) for the children in the richest quintile against that for the children from the lower four quintiles. Similarly, to examine the changes in maternal education gradient effect, we plot the smoothed values for HAZ/WAZ scores as a function of children’s age for the mothers’ who have attended secondary schooling against those who have no education or attended only primary school. The smoothed values in both instances are plotted with 95 % confidence interval.

Next, using multivariate Ordinary Least Squares (OLS) regressions, we examine age-specific socioeconomic gradients in child health. We divide the children into three age-specific groups: 0–11 months (year 0), 12–35 months (years 1–2), and 36–59 months (years 3–4). Given that growth faltering starts as early as in the first few months after birth [26], we consider the children in the 0–11 months group (year 0) as one group, and divide the older children into two equal groups. This allows us to examine if the age-specific gradients in socioeconomic measures emerge as early as in the first 11-months, and investigate if these gradient effects increase in the later age-groups.

We interact the socioeconomic status indicators with dummies representing child age groups [8, 15] and estimate the following two regression equations given in Eqs. (1) and (2) to identify the gradient effects. In both models, childhood health (CH) is the dependent variable; whereas household wealth status interacted with child age group dummies are the explanatory variables in Eq. (1), and maternal education replaces the household wealth in Eq. (2).

1 $$ {}{\fontsize{7.9pt}{9.3pt}\selectfont{\begin{aligned} \text{CH}=\alpha+\delta_{1}(\text{Richest Quintile})\times(\text{Age 0--11M}) + \delta_{2}(\text{Richest Quintile}) \\\times(\text{Age 12--35M}) + \delta_{3}(\text{Richest Quintile})\times(\text{Age 36--59M}) + X\beta+\epsilon \end{aligned}}}  $$

2 $$ {}{\fontsize{7.4pt}{9.3pt}\selectfont{ \begin{aligned} \text{CH}\,=\,\alpha\,+\,\gamma_{1}(\text{Maternal Education})\!\times\!(\text{Age 0--11M}) + \gamma_{2}(\text{Maternal Education}) \\\times(\text{Age 12--35M}) \,+\, \gamma_{3}(\text{Maternal Education})\!\times\!(\text{Age 36--59M})\! +\! X\beta\,+\,\epsilon \end{aligned}}}  $$

Because the dependent variables in both models are continuous in nature, we estimate the parameters of those equations using OLS. “Richest Quintile” in Eq. (1) presents a binary variable which takes a value 1 if the household belongs to the richest quintile, 0 if it belongs to the lower four quintiles. Likewise, “Maternal Education” variable takes a value 1 if the mother has attended secondary school, 0 otherwise. These categorizations follow Fernald et al. [3] who employ a similar categories of wealth and education while examining the gradient effects. Then, in both equations we use a set of control variables (X) that are discussed in the previous sub-section. *ε* is the random error term.

In Eqs. (1) and (2) above, the positive and significant coefficients of the interaction terms, (i.e., *δ*_1_, *δ*_2_, *δ*_3_> 0 in Eq. (1), and *γ*_1_, *γ*_2_, *γ*_3_>0 in Eq. (2)) provide evidence of age-specific gradients in household wealth and maternal education respectively. Further, a test of equality of the *δ* and *γ* coefficients will help us to examine if the gradients increase with age or not. In other words, this test of equality of coefficients will help us determine if the disparities in childhood health outcomes emerging from household wealth or maternal education attainment increase with age or not. The statistical analysis was conducted using STATA version 12.1. The “SVY” command was used to account for survey design and sampling weights to report the unbiased regression coefficients and the appropriate linearized standard errors.

Before presenting our results, it is important to address the potential presence of an endogenous household socioeconomic status measure in our model. Maternal education is highly unlikely to be affected by childhood health, and thus can be considered as exogenous. However, household wealth status can be potentially endogenous. Given that our analysis focuses on the children up to 59 months, it is unlikely that childhood health would directly contribute to household wealth via its effect on the earning potential of the child. However, child health may affect household wealth indirectly. The presence of chronically ill children may affect household wealth if parents have to sell household assets/properties to treat such children. Further, household wealth can be affected by parental labor market decisions which may be altered in the presence of a chronically ill child. In the presence of such an endogenous variable in the model, the interaction terms will be biased. This problem can be solved with the use of instrumental variables which should be correlated with the endogenous variable (household wealth status), but not with childhood health. We implemented an instrumental variable estimation using Lewbel’s method [27] that generates instruments as simple functions of the model’s data and followed that with endogenity tests similar to the Hausman test to examine if household wealth is endogenous to the model at all. Our finding suggested that household wealth can be safely treated as exogenous in the model, hence, the resulting interaction terms will be unbiased in the multivariate OLS estimation.

## Results

### Descriptive statistics

Table 1 presents the summary statistics for the variables used in this paper. The reported mean values and the corresponding standard deviations are very similar for most of the variables in both countries. The negative mean values of HAZ and WAZ scores indicate that larger proportions of children in both countries are stunted and underweight. A relatively larger proportion of children belongs to the poorest quintile in both Bangladesh (23.6 %) and Nepal (25.3 %). There are large discrepancies between both countries with respect to maternal educational attainment. While 20 % of mothers do not have any schooling in Bangladesh, this number is a staggering 46.9 % for Nepal. Similar observations can be made with respect to primary and secondary educational attainment where Bangladesh fairs better than Nepal. The only other visible difference is that nearly 91 % of the children reside in rural areas in Nepal where as it is relatively lower at 78 % in Bangladesh. Tables S1 and S2 in the Additional file 1 present the sample characteristics by wealth quintiles and maternal education categories for Bangladesh and Nepal, respectively.

**Table 1:** Summary statistics

Variable	Variable definition	Bangladesh		Nepal	
		Mean	Std. Dev.	Mean	Std. Dev.
HAZ	Height for Age Z Score	–1.673	1.404	–1.656	1.389
WAZ	Weight for Age Z Score	–1.601	1.154	–1.418	1.096
Child Specific Variables					
Child’s Age Group					
Age 0–11M	1 if child is in 0–11 months age group, otherwise 0	0.197	0.398	0.196	0.397
Age 12–35M	1 if child is in 12–35 months age group, otherwise 0	0.374	0.484	0.404	0.491
Age 36–59M	1 if child is in 36–59 months age group, otherwise 0	0.429	0.495	0.400	0.490
Male	1 if child is a boy, otherwise 0	0.510	0.500	0.506	0.500
Birth Size	1 if child’s size at birth is below average, otherwise 0	0.170	0.376	0.178	0.382
Birth Order	Child’s birth order	2.395	1.568	2.556	1.841
Mother Specific Variables					
Mother’s Age	Current age of mother (in years)	25.531	5.885	26.758	5.996
Maternal Education					
No Education	1 if mother does not have any education, otherwise 0	0.201	0.401	0.469	0.499
Primary	1 if mother has attended primary education, otherwise 0	0.305	0.461	0.198	0.398
Secondary	1 if mother has attended secondary or higher education, otherwise 0	0.493	0.500	0.332	0.471
Mother’s Height	Mother’s height (in centimeters)	150.895	5.465	151.122	5.315
Mother’s BMI	Mother’s body mass index	20.754	3.428	20.976	2.937
Father Specific Variables					
Father’s Age	Current age of father (in years)	34.218	7.905	31.017	7.299
Father’s Education					
No Education	1 if father does not have any education, otherwise 0	0.296	0.457	0.219	0.414
Primary	1 if father has attended primary education, otherwise 0	0.291	0.454	0.246	0.431
Secondary	1 if father has attended secondary or higher education, otherwise 0	0.412	0.492	0.535	0.499
Household Specific Variables					
Household Wealth Status					
Richest	1 if household is in the richest quintile, otherwise 0	0.176	0.381	0.139	0.346
Richer	1 if household is in the richer quintile, otherwise 0	0.189	0.392	0.172	0.378
Middle	1 if household is in the middle quintile, otherwise 0	0.196	0.397	0.234	0.424
Poorer	1 if household is in the poorer quintile, otherwise 0	0.204	0.403	0.202	0.401
Poorest	1 if household is in the poorest quintile, otherwise 0	0.236	0.424	0.253	0.435
Log (Household Size)	Log of Household Size	1.735	0.397	1.731	0.412
Rural	1 if it is a rural household, otherwise 0	0.780	0.414	0.911	0.284
Religion	1 if a Muslim (Hindu) household in Bangladesh (Nepal), otherwise 0	0.913	0.281	0.838	0.368
Weighted Sample Size		7505		2342	

### Graphical analysis

**Fig. 1:**
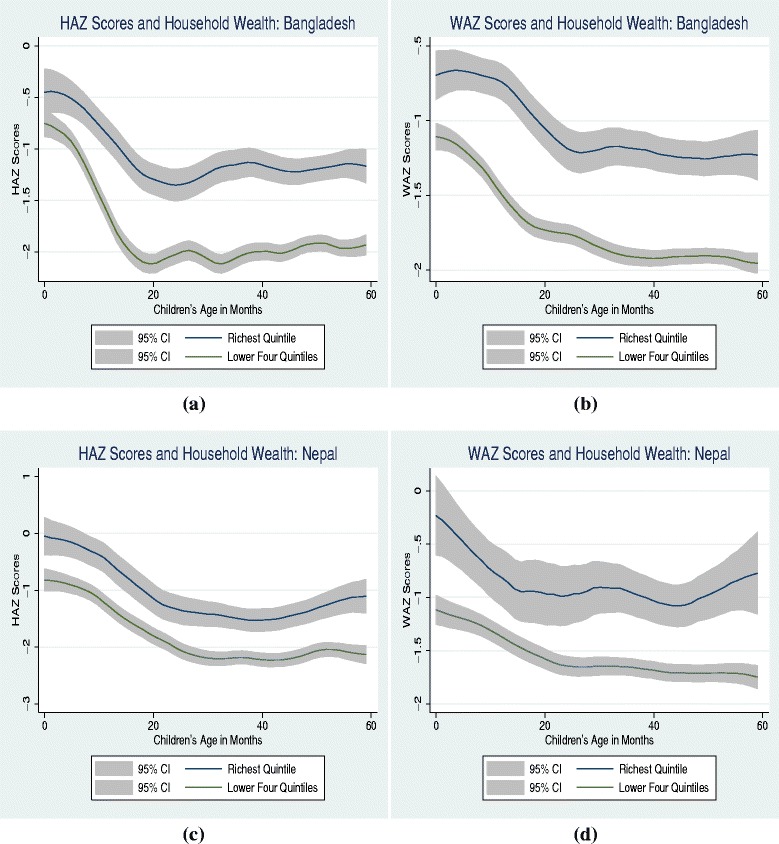
Early childhood health and household wealth

**Fig. 2:**
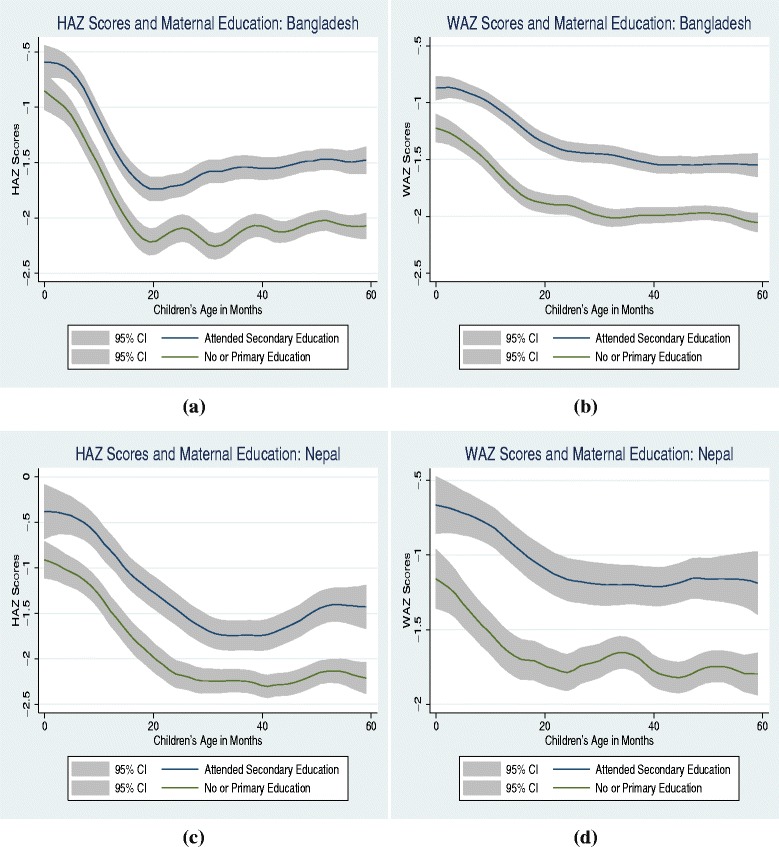
Early childhood health and maternal education

Figures 1 and 2 provide evidence of household wealth and maternal education gradients in child health respectively. Children from the richest quintile display significantly higher health outcomes in comparison to those from the lower four quintiles in both countries. Similarly, children of mothers who have attended secondary education experience significant health advantages in comparison to those of with lower educational attainment. Health disparities in socioeconomic status emerge as early as in the first month of life and increase through the early childhood in most cases in both countries.^2^ However, this analysis does not control for additional confounding factors, hence, may display an incomplete picture on socioeconomic gradients.

### Regression results

Table 2 presents the regression estimates from the model presented in Eq. (1) and examines the gradients emerging from household wealth. We limit the presentation of results only to gradient effects to address the objectives of the study concisely. The first two columns present the results for Bangladesh, and the last two columns for Nepal. The first three rows present the estimated coefficients on the interaction terms between household wealth status and age-group indicators, and the other rows present the estimated coefficients on other control variables. The coefficients of the interaction terms are positive and significant in each age-group in both Column 1 and Column 2 when we use HAZ and WAZ scores as the dependent variables, respectively. These results provide significant evidence on the presence of age-specific gradient effects in Bangladesh. However, the coefficients of the interaction terms are not statistically different from each other in both columns and suggest that wealth gradient in health does not increase with age. On the contrary, the interaction terms are positive and insignificant in Nepal irrespective of the choice of the dependent variable (HAZ scores in Column 3 and WAZ scores in Column 4). These findings suggest a lack of wealth gradient effect in early childhood health in Nepal.

**Table 2:** Age-specific household wealth gradients in early childhood health

	Bangladesh		Nepal	
	(1)	(2)	(3)	(4)
	HAZ	WAZ	HAZ	WAZ
Age 0–11M ×Richest Q.	0.271**	0.243***	0.171	0.043
	(0.113)	(0.087)	(0.171)	(0.147)
Age 12–35M ×Richest Q.	0.465***	0.346***	0.255	0.140
	(0.084)	(0.071)	(0.155)	(0.119)
Age 36–59M ×Richest Q.	0.372***	0.210***	0.071	0.182
	(0.074)	(0.068)	(0.131)	(0.127)
Child specific controls				
Child’s age				
Age 0–11 M (Reference)				
Age 12–35M	–1.035***	–0.581***	–0.951***	–0.344***
	(0.052)	(0.042)	(0.094)	(0.081)
Age 36–59M	–1.007***	–0.784***	–1.073***	–0.470***
	(0.053)	(0.043)	(0.103)	(0.089)
Male	–0.003	0.051*	–0.057	–0.067
	(0.033)	(0.027)	(0.060)	(0.049)
Birth size	–0.347***	–0.458***	–0.341***	–0.415***
	(0.046)	(0.037)	(0.080)	(0.055)
Birth order	–0.114***	–0.075***	–0.058*	–0.092***
	(0.021)	(0.016)	(0.030)	(0.025)
Maternal controls				
Mother’s age	0.033***	0.018***	0.013	0.015
	(0.006)	(0.005)	(0.011)	(0.009)
Mother’s education				
No education (Reference)				
Primary education	0.074	0.044	0.087	0.126
	(0.058)	(0.046)	(0.101)	(0.077)
Secondary education	0.172***	0.223***	0.248**	0.328***
	(0.060)	(0.050)	(0.102)	(0.083)
Mother’s height	0.056***		0.058***	
	(0.003)		(0.007)	
Mother’s BMI		0.064***		0.067***
		(0.005)		(0.010)
Paternal controls				
Father’s age	0.003	0.001	–0.007	–0.001
	(0.003)	(0.003)	(0.008)	(0.006)
Father’s education				
No Education (Reference)				
Primary education	–0.011	–0.032	0.092	0.052
	(0.049)	(0.043)	(0.130)	(0.099)
Secondary education	0.199***	0.091**	0.178	0.091
	(0.055)	(0.045)	(0.127)	(0.094)
Household controls				
Log (Household size)	0.005	0.032	0.029	0.000
	(0.045)	(0.039)	(0.102)	(0.070)
Rural	–0.026	–0.019	–0.289***	–0.067
	(0.052)	(0.045)	(0.102)	(0.076)
Religion	–0.120*	–0.119**	–0.160	–0.175***
	(0.062)	(0.054)	(0.100)	(0.067)
Weighted Sample Size	7505	7504	2342	2339
R-Squared	0.204	0.199	0.210	0.180
F	55.593	50.682	21.665	15.466
Mean VIF	2.17	2.18	1.76	1.77

Note: The numbers in the parenthesis refer to the linearized standard errors that account for survey design and sample weight. ***indicates *p*< 0.01, ** indicates *p* < 0.05 and *indicates *p* <0.1. Each regression also includes dummies to capture regional effects. The regional dummies for Bangladesh are based on the seven major divisions, and for Nepal the regional dummies are based on the five developmental regions. The religion dummy captures the Muslim and Hindu religions for Bangladesh and Nepal respectively

To examine if the wealth gradient effect is driven by a particular wealth quintile, we run a slightly modified version of the model presented in Eq. (1). In this modified model, we interact dummies for each wealth quintile with child age group indicators [15]. Contrary to Table 2, we consider the richest quintile as the reference wealth category in these regressions. Given that children from the lower quintiles are expected to have lower health outcomes in comparison to the richest one, we expect the coefficients of the interactions terms to be negative. These results are reported in Table 3. This table only reports the coefficients of interaction terms and the full set of results including those for control variables, are available on request. Each column of the table presents the wealth quintile indicators and each row presents the age-group indicators. Hence, each cell in the cross-section presents the corresponding interaction term. In Panel A, the coefficients of interaction terms are negative and significant except the interaction term for richer quintile and 0–11 months age-group when we use HAZ scores as the dependent variable for Bangladesh. Similarly, with the WAZ scores as the dependent variable for Bangladesh in Panel B, interaction coefficients are negative and significant except the interaction terms for 0–11 months age group, and richer and middle quintiles. Further, the strength of the interaction coefficients usually improves as we move from the poorest quintile towards the richer quintile in each age group. This implies that the disparity in terms of lower HAZ/WAZ scores is the strongest for the children from the poorest quintile across age groups.

**Table 3:** Age-specific household wealth gradients in early childhood health (By Wealth Quintiles)

	Panel A			
	Bangladesh (Dependent Variable: HAZ Scores)			
	Poorest	Poorer	Middle	Richer
Age 0–11 M	–0.579***	–0.451***	–0.250*	–0.157
	(0.146)	(0.132)	(0.134)	(0.136)
Age 12–35 M	–0.749***	–0.522***	–0.485***	–0.456***
	(0.105)	(0.107)	(0.099)	(0.094)
Age 36–59 M	–0.643***	–0.513***	–0.428***	–0.283***
	(0.100)	(0.090)	(0.088)	(0.087)
	Weighted Sample: 7505, R-Squared: 0.209; F: 43.48, Mean VIF: 2.83			
	Panel B			
	Bangladesh (Dependent Variable: WAZ Scores)			
	Poorest	Poorer	Middle	Richer
Age 0–11 M	–0.581***	–0.373***	–0.159	–0.105
	(0.109)	(0.109)	(0.102)	(0.112)
Age 12–35 M	–0.573***	–0.430***	–0.330***	–0.302***
	(0.087)	(0.086)	(0.084)	(0.081)
Age 36–59 M	–0.320***	–0.283***	–0.322***	–0.217***
	(0.085)	(0.080)	(0.082)	(0.076)
	Weighted Sample: 7504, R-Squared: 0.205; F: 38.98, Mean VIF: 2.85			
	Panel C			
	Nepal (Dependent Variable: HAZ Scores)			
	Poorest	Poorer	Middle	Richer
Age 0–11 M	–0.503**	–0.659***	–0.018	–0.070
	(0.226)	(0.222)	(0.207)	(0.238)
Age 12–35 M	–0.649***	–0.396**	–0.247	–0.113
	(0.176)	(0.180)	(0.207)	(0.177)
Age 36–59 M	–0.384**	–0.228	–0.165	0.054
	(0.161)	(0.178)	(0.178)	(0.169)
	Weighted Sample: 2342, R-Squared: 0.224; F: 17.24, Mean VIF: 2.89			
	Panel D			
	Nepal (Dependent Variable: WAZ Scores)			
	Poorest	Poorer	Middle	Richer
Age 0–11 M	–0.130	–0.532**	0.061	0.074
	(0.194)	(0.222)	(0.180)	(0.194)
Age 12–35 M	–0.344**	–0.254*	–0.199	0.001
	(0.141)	(0.146)	(0.164)	(0.127)
Age 36–59 M	–0.214	–0.310**	–0.371**	–0.118
	(0.154)	(0.154)	(0.155)	(0.145)
	Weighted Sample: 2339, R-Squared: 0.193; F: 11.78, Mean VIF: 2.90			

Note: The numbers in the parenthesis refer to the linearized standard errors that account for survey design and sample weight. ***indicates *p*< 0.01, **indicates *p* < 0.05 and *indicates *p* <0.1. Each panel presents a separate regression. The table reports regression coefficients of the interaction terms of dummies for each wealth quintile with dummies for child age groups where the richest quintile is the reference category. We control for same set of control variables as mentioned in Table 2. The regressions for HAZ scores control for the same set of control variables as displayed in Table 2 columns (1) and (3) for Bangladesh and Nepal respectively. The regressions for WAZ scores control for the same set of control variables as displayed in Table 2 columns (2) and (4) for Bangladesh and Nepal respectively. Each regression also includes dummies to capture regional effects. The regional dummies for Bangladesh are based on the seven major divisions, and for Nepal the regional dummies are based on the five developmental regions

Tests of equality of the coefficients of interaction terms across each quintile suggest that estimated coefficients are not statistically different. The only exceptions are richest quintile in Panel A (HAZ Scores) and the poorest quintile in Panel B (WAZ Scores). The coefficient of the interaction term for the richest quintile increases between 0–11 month and 12–35 month groups in Panel A. On the contrary, the wealth gradient in WAZ scores reduces (statistically significantly) after 35 months for the children from the poorest quintile.

Panels C and D in Table 3 present the coefficients of the interaction terms from regression models for Nepal with HAZ and WAZ scores as the dependent variables, respectively. The interaction coefficients are though negative, are only significant for the bottom two quintiles in Panel C. These results suggest that the children from the bottom two quintiles experience significantly lower HAZ scores in comparison to the richest quintile.^3^ Similarly, the interaction coefficients are mostly negative and significant only for the bottom two quintiles in Panel D with WAZ scores as the dependent variable.^4^ Importantly, Table 3 reveals that in contrast to Table 2, wealth gradients in early childhood health exist in Nepal, albeit only for the bottom two quintiles. However, interaction coefficients do not increase (statistically) with age for the bottom two wealth quintiles.

Table 4 presents the evidence on age-specific maternal education gradients in childhood health from the OLS regressions that follow Eq. (2). The first three rows of the table present the estimated coefficients on the interaction terms between maternal education and age-group indicators, and the other rows present the estimated coefficients on other control variables. The literature examining maternal education gradients in child health usually does not control for father’s education [3]. To be consistent, we present two sets of results in Table 4. While the odd-numbered columns present the coefficients from models that exclude the control variables on father’s education, the adjacent even-numbered columns reproduce the estimates from identical models with father’s education as the additional control variable. Column 1 presents the results for the regression model with HAZ scores as the dependent variable for Bangladesh. The coefficients of the interaction terms are positive and significant for the later two age groups though not statistically different from each other. However, the coefficients of the interaction terms are positive and significant for all the three age groups for Bangladesh when WAZ scores are used as the dependent variable (Column 3). Tests of equality of coefficients suggest that the interaction terms are statistically different from each other in the later two age groups implying that the effect of maternal education on WAZ scores declines after 35 months. In contrast, significant maternal education gradient in HAZ scores is observed only for the 12–35 months age group in Nepal (Column 5). On the other hand, interaction terms are positive and significant in each age group in Nepal with WAZ scores as the dependent variable (Column 7). These effects are not statistically different across age-groups. Interestingly, the strength of maternal education gradients either declines or completely disappears across the board after controlling for father’s education in the even-numbered columns.

**Table 4:** Age-specific maternal education gradients in early childhood health

	Bangladesh				Nepal			
	(1)	(2)	(3)	(4)	(5)	(6)	(7)	(8)
	HAZ	HAZ	WAZ	WAZ	HAZ	HAZ	WAZ	WAZ
Age 0–11M × Mothers with Sec.Educ.	0.003	–0.038	0.186***	0.168**	0.106	0.092	0.240*	0.232
	(0.086)	(0.086)	(0.067)	(0.068)	(0.167)	(0.171)	(0.140)	(0.141)
Age 12–35M × Mothers with Sec.Educ.	0.139**	0.095	0.268***	0.247***	0.247**	0.225*	0.277***	0.266**
	(0.066)	(0.065)	(0.053)	(0.054)	(0.124)	(0.126)	(0.104)	(0.104)
Age 36–59M × Mothers with Sec.Educ.	0.122**	0.079	0.078*	0.060	0.089	0.063	0.201**	0.182*
	(0.052)	(0.055)	(0.047)	(0.049)	(0.103)	(0.110)	(0.097)	(0.098)
Child specific controls								
Child’s age								
Age 0–11 M (Reference)								
Age 12–35 M	–1.073***	–1.067***	–0.604***	–0.600***	–0.996***	–0.992***	–0.351***	–0.349***
	(0.070)	(0.070)	(0.057)	(0.057)	(0.104)	(0.109)	(0.098)	(0.100)
Age 36–59 M	–1.052***	–1.048***	–0.738***	–0.737***	–1.094***	–1.089***	–0.447***	–0.439***
	(0.069)	(0.069)	(0.053)	(0.053)	(0.107)	(0.107)	(0.101)	(0.101)
Male	–0.007	–0.005	0.050*	0.052*	–0.040	–0.048	–0.059	–0.063
	(0.034)	(0.034)	(0.027)	(0.027)	(0.059)	(0.059)	(0.048)	(0.048)
Birth size	–0.345***	–0.342***	–0.454***	–0.452***	–0.344***	–0.328***	–0.420***	–0.413***
	(0.046)	(0.046)	(0.037)	(0.037)	(0.081)	(0.078)	(0.055)	(0.054)
Birth order	–0.109***	–0.103***	–0.070***	–0.068***	–0.052*	–0.050*	–0.090***	–0.089***
	(0.021)	(0.021)	(0.017)	(0.017)	(0.031)	(0.030)	(0.026)	(0.025)
Maternal controls								
Mother’s age	0.031***	0.031***	0.017***	0.016***	0.018	0.016	0.017*	0.016*
	(0.006)	(0.006)	(0.005)	(0.005)	(0.012)	(0.011)	(0.009)	(0.009)
Mother’s height	0.056***	0.056***			0.058***	0.057***		
	(0.003)	(0.003)			(0.006)	(0.007)		
Mother’s BMI			0.062***	0.062***			0.069***	0.068***
			(0.005)	(0.005)			(0.010)	(0.010)
Paternal controls								
Father’s age	0.003	0.003	0.001	0.000	–0.011	–0.009	–0.005	–0.003
	(0.003)	(0.003)	(0.003)	(0.003)	(0.008)	(0.008)	(0.006)	(0.006)
Father’s education								
No education (Reference)								
Primary education		–0.044		–0.054		0.082		0.064
		(0.048)		(0.041)		(0.126)		(0.099)
Secondary education		0.129**		0.046		0.118		0.084
		(0.057)		(0.044)		(0.125)		(0.097)
Household characteristics								
Household wealth status								
Richest	0.736***	0.677***	0.496***	0.472***	0.537***	0.506***	0.264**	0.243**
	(0.075)	(0.079)	(0.062)	(0.064)	(0.131)	(0.130)	(0.114)	(0.118)
Richer	0.395***	0.355***	0.256***	0.242***	0.502***	0.472***	0.243***	0.220**
	(0.063)	(0.067)	(0.056)	(0.057)	(0.121)	(0.118)	(0.093)	(0.100)
Middle	0.287***	0.262***	0.182***	0.174***	0.364***	0.352***	0.051	0.041
	(0.059)	(0.060)	(0.045)	(0.045)	(0.115)	(0.113)	(0.085)	(0.086)
Poorer	0.178***	0.171***	0.109**	0.109**	0.126	0.138	-0.075	-0.074
	(0.055)	(0.055)	(0.044)	(0.044)	(0.120)	(0.121)	(0.090)	(0.092)
Poorest (Reference)								
Log (Household Size)	–0.043	–0.051	–0.001	–0.006	–0.009	–0.004	–0.029	–0.028
	(0.045)	(0.045)	(0.040)	(0.040)	(0.096)	(0.098)	(0.065)	(0.066)
Rural	0.043	0.038	0.028	0.025	-0.227**	-0.230**	-0.030	-0.029
	(0.052)	(0.052)	(0.046)	(0.046)	(0.106)	(0.106)	(0.079)	(0.079)
Religion	–0.136**	–0.128**	–0.129**	–0.124**	–0.177*	–0.175*	–0.190***	–0.191***
	(0.062)	(0.062)	(0.055)	(0.055)	(0.100)	(0.102)	(0.067)	(0.068)
Weighted Sample Size	7508	7505	7508	7504	2352	2342	2349	2339
R-squared	0.206	0.208	0.202	0.203	0.223	0.221	0.186	0.185
F	56.845	52.922	51.842	47.706	26.393	22.717	15.362	14.045
Mean VIF	2.34	2.34	2.36	2.36	1.84	1.89	1.85	1.90

Note: The numbers in the parenthesis refer to the linearized standard errors that account for survey design and sample weight. ***indicates *p*< 0.01, **indicates *p* < 0.05 and *indicates *p* <0.1. Each regression also includes dummies to capture regional effects. The regional dummies for Bangladesh are based on seven major divisions and for Nepal, the regional dummies are based on five developmental regions. The religion dummy captures the Muslim and Hindu religions for Bangladesh and Nepal respectively

Analogous to the exercise with respect to wealth gradient (Table 3), we also run additional regressions to examine if the maternal education effect is resulting from a particular educational category. As opposed to Table 4, we consider the secondary schooling as the reference category, and interact dummies for other two maternal educational categories (primary education and no education) with child age group indicators. Given that we expect the children from mothers with less education to experience lower health outcomes, we expect the coefficients of these interaction terms to be negative. These regression results are reported in Table S3 in the Additional file 1. We only report the coefficients for the interaction terms in this table. These results do not provide any additional insights with respect to the emergence and changes in maternal education gradient effects across age-groups though the gradient effect is stronger for children of mother’s without any education. Specifically, children in the lowest educational category in Nepal display significant health disadvantages in HAZ/WAZ scores in comparison to those from primary or secondary education categories.

## Discussion

This paper examines socioeconomic gradients in early childhood health using the DHS data from Bangladesh and Nepal. This section discusses our results and their implications for the existing public policy discourse in four specific points.

First, household wealth emerges as an important determinant of early childhood health while controlling for a rich set of confounding factors. Children from the richest quintile experience significantly higher HAZ/WAZ scores in comparison to those from the bottom four quintiles in each age-group in Bangladesh. This disparity in terms of lower health status is strongest for children from the poorest quintile. Similarly, significant wealth gradients in health are observed across age-groups in Nepal, though only in the bottom two quintiles. Unlike studies from Mozambique [11] and China [14] that identify gradients in economic resources only among children older than 2 years, we observe the wealth gradients in both countries as early as the first 11 months. This finding reconciles with the literature that identifies faltering growth in children in the first few months after birth [26], and further reveals that these adverse growth outcomes are more pronounced in children from the lower economic strata in both countries. Our finding emphasizes that household wealth status is an important risk factor for early childhood health and draws support from the previous studies on Bangladesh [28–32] and Nepal [33, 34].

Second, children of mothers with secondary education display significantly higher health outcomes in both countries, a finding consistent with earlier studies from Bangladesh [30–32] and Nepal [33, 34]. While the effect of maternal education on WAZ scores emerges within the first 11 months of life, its effect on HAZ scores is only observed among children older than 11 months in both countries. Recall that HAZ scores represent faltering growth resulting from longer-term deficiency in dietary intake. As larger proportions of children are exclusively breastfed through the first 6 months in both countries [23, 24], maternal education may play an important role as a child grows older when the introduction and continuance of appropriate complementary food practices play a greater role in determining child’s long-term nutritional status. In contrast, WAZ scores reflect both short and long-term health status, and can be affected by short spells of acute infections or fever even in very early months of life. Children of mothers with higher education may recover sooner and display higher WAZ scores due to such mothers’ knowledge and awareness on superior health care practices. Evidence from Bangladesh and Nepal suggests that educated mothers are more likely to seek health care services in case of onsets of acute infections [23, 24]. Further, the effect of maternal education on childhood health is not uniform across age groups in many instances, and certainly not increasing with age in both countries. For example, the effect of maternal education on HAZ scores disappears for the children in the 36–59 months group in Nepal. It could be argued that maternal education is more important in the early years life in Nepal given the care required. Studies on Mozambique [11] and Indonesia [12] also report similar findings.

Interestingly, the strength of maternal education gradients decreases or disappears altogether in both countries once the model controls for father’s education. This might be explained by the fact that both Bangladesh and Nepal are patriarchal societies, and typically, the father commands a greater role in the decision making on household’s access to health care and nutrition [35, 36]. Public health research is rich with studies that document such findings in the context of developing countries [37].

Third, the observed wealth and maternal education gradient effects do not increase with age in most instances, though they reduce in strength or disappear in few cases among older children. These outcomes may be subject to bias resulting from infant and child mortality rates. If children from lower socioeconomic statuses experience higher mortality rates, it will artificially raise the average health status of these children. Furthermore, if these mortality rates have declined over time, the average health of older children from this group will be even higher [12, 15]. The presence of such biases will not only weaken the socioeconomic gradients in health, but also it will make it harder to observe stronger gradient effects among older children. Evidence from Bangladesh and Nepal gives credence to both these arguments [23, 24]. However, given that the infant and under-five mortality rates for Bangladesh (43 and 53) [23] and Nepal (46 and 54) [24] are marginally higher than the world average of 35 and 47 per thousand live births [38], it is difficult to speculate to what extent these mortality rates really affect our results.

As a supplemental argument, we mostly find the gradients in WAZ scores to reduce in strength or disappear among older children. Recall that WAZ scores can get affected by short spells of acute infections (such as diarrhea and acute respiratory infections) and fever. Prevalence of such conditions usually declines as children gets older in both countries [23, 24]. This might potentially explain our finding of weaker or a lack of gradient effect in WAZ scores among children in the oldest age-group.

Fourth, our findings have important public policy implications for both countries as lower early childhood health outcomes have ramifications for future health and education attainment, and labor market performance [20]. The existence of health inequalities in household wealth demands targeted policy interventions in the form of food security and nutrition supplement programs, and free provision of health care access, specifically for the children in the poorest strata and in early months of life. Food security and nutrition programs have been central to the public policies initiated in Bangladesh and Nepal. For example, the government of Bangladesh has introduced the National Nutrition Service (NNS) program for 2011–2016 in order to reduce child and maternal malnutrition, focusing especially on the underprivileged strata of the country. However, implementation and coverage of such policies have been weak to date [39]. Similarly, the National Planning Commission of Nepal has developed a Multi-Sectoral Nutrition Plan (MSNP) in 2012 to improve maternal and child nutrition [40]. Our findings suggest that the effective implementation of such programs to target the poorest sections of the society will be critical to address the inception of economic disparities in child health in early years.

Similarly, active initiatives must be undertaken to reduce health inequalities resulting from maternal educational attainment. Bangladesh has achieved remarkable success in female educational attainment owing to the conditional cash transfer program initiated in 1994, known as the Female Secondary Stipend (FSS) program [41]. On the other hand, maternal educational attainment is extremely poor in Nepal with 47 % of mothers with no education. While Bangladesh should continue with the successful implementation of the FSS program, Nepal should initiate similar cash transfer programs to improve both primary and secondary female enrollment rates. Further, initiatives also must be pursued in both countries to improve male educational attainment as well given that the effect of maternal education on child’s health reduces in the presence of an educated father.

**Limitations:** This study has a few limitations. First, we use household wealth as a measure of economic status instead of income or consumption. While wealth represents the long-term economic standing of a household, income and consumption address immediate household needs of food and health care, and hence, may be stronger predictors of childhood health. The lack of data on income and consumption in the DHS limits us to explore this linkage. However, the use of household wealth is also common in the literature on socioeconomic gradients in childhood health [3, 12]. Secondly, our study is limited to children up to 59 months due to the lack of data on health measures for older children in the DHS. This restricts our analysis to the changes that take place after 59-months as done in few other studies on developing countries [13, 14]. Finally, our current analysis relies on cross-section surveys from 2011. Thus, we can not track changes in the age-specific socioeconomic gradients over time.

## Conclusions

This study examines socioeconomic gradients in early childhood health in Bangladesh and Nepal using the 2011 Demographic and Health Surveys. The paper provides significant evidence of age-specific socioeconomic gradients in health in both countries. Health disparities in household wealth exist in both countries. Specifically, the disparity in terms of lower health outcomes is strongest for the children from poorest quintile. This disparity emerges in the first 11 months of age. On the other hand, while the timing of the emergence of maternal education gradients is sensitive to the choice of childhood health measure, the study finds that children of mothers with higher education enjoy significant health advantages in comparison to those of with lower education. However, controlling for father’s education weakens the strength of maternal education gradients in both countries. Further, the study does not find household wealth and maternal education gradient effects to increase with age. The bias resulting from infant and child mortality rates may be a potential source of this outcome. Nevertheless, our study shows that socioeconomic disparities in health exist in early childhood. This has important policy implications for food security and nutrition supplement programs, free provision of health care access, and female education in both countries.

## Ethics statement

The 2011 Bangladesh DHS was approved by the National Research Ethics Committee of the Bangladesh Medical Research Council, and by the ICF Macro Institutional Review Board. The 2011 Nepal DHS was approved by the Nepal Health Research Council Ethical Review Board. The 2011 Nepal DHS categorized under the MEASURE DHS Project Phase III, was approved by ICF Macro. The Institutional Review Board of ICF Macro complied with the United States Department of Health and Human Services requirements for the “Protection of Human Subjects” (45 CFR 46).

## Endnotes

^1^ The weighted sample size for Bangladesh is 7504, and 2339 in Nepal if we use mother’s BMI as an explanatory variable in the model.

^2^ This evidence is relatively weaker in few instances, such as Figs. 1c and 2b.

^3^ The coefficient of the interaction term for the 36–59 months age group and the poorer quintile is insignificant.

^4^ The coefficient for 36–59 months age group in the middle quintile is negative and significant. The strength of this coefficient is slightly larger in absolute value in comparison to those from the bottom two quintiles for the same age group. Also, the coefficients for 0–11 months and 36–59 months age groups in the poorest quintile are insignificant.

## Additional file

Additional file 1 Supplementary figures and tables. (PDF 72 kb)

## Abbreviations

BMI: body mass index
DHS: demographic and health surveys
FSS: female secondary stipend
HAZ: height-for-age Z-score
MSNP: multi-sectoral nutrition plan
NNS: national nutrition service
OLS: ordinary least squares
USAID: U.S. Agency for international development
WAZ: weight-for-age Z-score
