# Supplementary material for: Socioeconomic gradients in early childhood health: evidence from Bangladesh and Nepal
Source: Int J Equity Health. 2016 May 16;15:78. doi: 10.1186/s12939-016-0364-2 (PMC4867530; doi:10.1186/s12939-016-0364-2)
Supplement: Additional file 1 — Supplementary figures and tables. (PDF 72 kb) [file 12939_2016_364_MOESM1_ESM.pdf]

Socioeconomic Gradients in Early Childhood Health: Evidence from Bangladesh and Nepal (Supplementary Figures and Tables)

Satis Devkota* Bibhudutta Panda†

April 9, 2016

*University of Minnesota-Morris, Division of Social Science, 600 East 4th Street, Morris, MN 56267. Phone: 320-589-6198, Email: sdevkota@morris.umn.edu

†Corresponding Author: University of Minnesota-Morris, Division of Social Science, 600 East 4th Street, Morris, MN 56267. Phone: 320-589-6205, Email: bpanda@morris.umn.edu

Figure S1: **Flow Chart of Sample Selection**

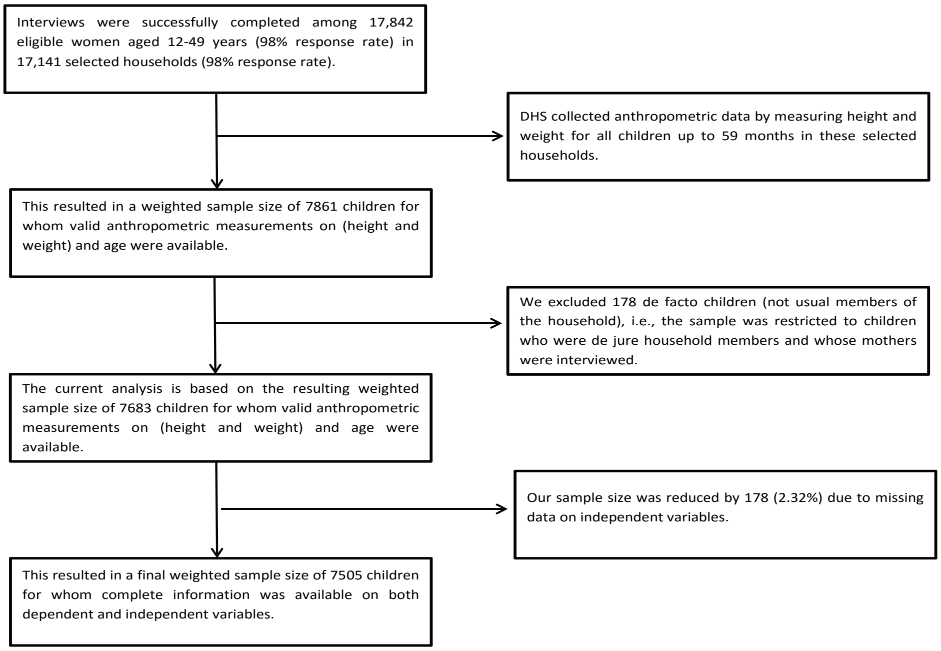

(a) Bangladesh

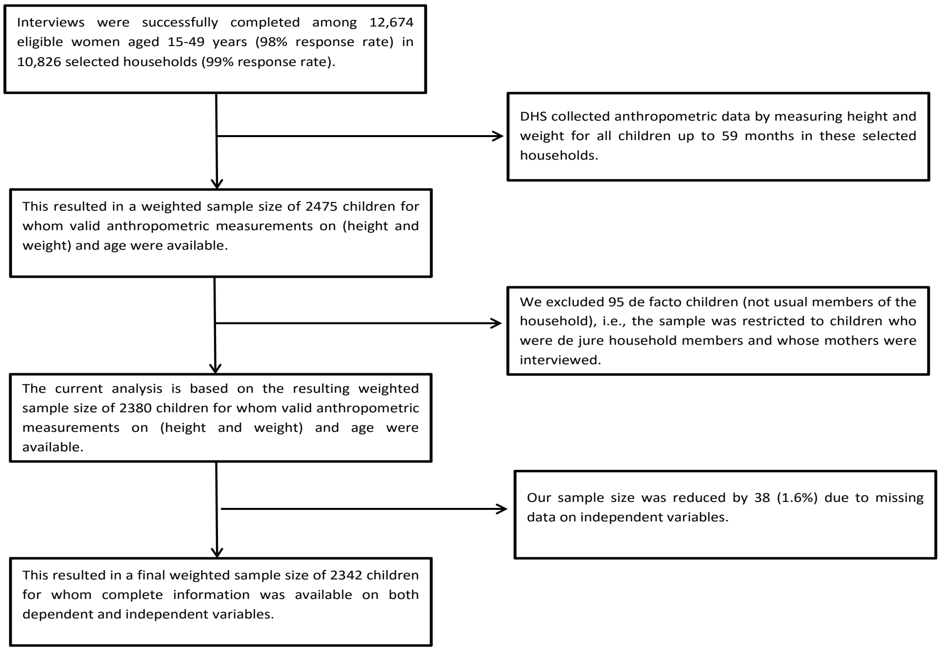

(b) Nepal

Table S1: Sample Characteristics by Wealth and Maternal Education Categories (Bangladesh)

	Wealth Quintiles					Mother's Education		
	Poorest	Poorer	Middle	Richer	Richest	No Educ.	Primary	Secondary
	Mean (SD)					Mean (SD)		
HAZ	-2.111	-1.828	-1.678	-1.511	-1.074	-1.998	-1.864	-1.422
	(1.356)	(1.285)	(1.297)	(1.364)	(1.522)	(1.377)	(1.374)	(1.387)
WAZ	-1.989	-1.777	-1.616	-1.432	-1.044	-1.920	-1.802	-1.348
	(1.045)	(1.017)	(1.087)	(1.109)	(1.324)	(1.052)	(1.086)	(1.180)
Child Specific Variables								
Child's Age Group								
Age 0-11M	0.186	0.191	0.199	0.215	0.197	0.165	0.187	0.216
	(0.375)	(0.384)	(0.395)	(0.416)	(0.425)	(0.360)	(0.388)	(0.418)
Age 12-35M	0.371	0.378	0.375	0.361	0.387	0.334	0.377	0.389
	(0.467)	(0.474)	(0.479)	(0.487)	(0.520)	(0.457)	(0.483)	(0.494)
Age 36-59M	0.443	0.431	0.426	0.424	0.416	0.501	0.437	0.394
	(0.479)	(0.484)	(0.489)	(0.501)	(0.526)	(0.485)	(0.494)	(0.496)
Male	0.496	0.511	0.523	0.513	0.512	0.497	0.524	0.508
	(0.482)	(0.488)	(0.494)	(0.507)	(0.534)	(0.485)	(0.498)	(0.507)
Birth Size	0.190	0.186	0.174	0.152	0.141	0.183	0.175	0.162
	(0.379)	(0.380)	(0.375)	(0.364)	(0.371)	(0.375)	(0.379)	(0.373)
Birth Order	2.958	2.517	2.276	2.166	1.877	3.651	2.529	1.800
	(1.733)	(1.664)	(1.494)	(1.365)	(1.080)	(1.966)	(1.480)	(0.980)
Mother Specific Variables								
Mother's Age	26.104	25.235	25.090	25.166	25.992	29.080	25.209	24.284
	(5.964)	(6.004)	(5.899)	(5.555)	(5.749)	(6.635)	(5.532)	(5.100)
Maternal Education								
No Education	0.436	0.238	0.128	0.090	0.045	-	-	-
	(0.478)	(0.416)	(0.330)	(0.290)	(0.222)			
Primary	0.395	0.389	0.320	0.257	0.125	-	-	-
	(0.472)	(0.476)	(0.462)	(0.443)	(0.353)			
Secondary	0.169	0.373	0.552	0.654	0.830	-	-	-
	(0.362)	(0.472)	(0.492)	(0.482)	(0.401)			
Mother's Height	149.877	150.881	150.749	151.172	152.143	150.046	150.325	151.594
	(5.540)	(5.298)	(5.286)	(5.336)	(5.539)	(5.274)	(5.495)	(5.430)
Mother's BMI	19.261	19.915	20.441	21.588	23.183	19.917	20.103	21.498
	(2.494)	(2.589)	(3.050)	(3.562)	(4.181)	(2.887)	(3.039)	(3.704)
Father Specifc Variables								
Father's Age	34.818	33.799	33.518	34.062	34.845	38.490	33.707	32.793
	(8.277)	(8.178)	(7.765)	(7.408)	(7.390)	(8.892)	(7.828)	(6.770)
Father's Education								
No Education	0.586	0.376	0.233	0.137	0.058	0.666	0.367	0.102
	(0.475)	(0.473)	(0.418)	(0.348)	(0.249)	(0.458)	(0.481)	(0.307)
Primary	0.312	0.362	0.318	0.278	0.165	0.238	0.407	0.242
	(0.447)	(0.469)	(0.461)	(0.454)	(0.396)	(0.413)	(0.490)	(0.434)
Secondary	0.102	0.261	0.449	0.585	0.777	0.096	0.226	0.656
	(0.292)	(0.429)	(0.492)	(0.499)	(0.444)	(0.286)	(0.417)	(0.482)
Hosuehold Specific Variables								
Household Wealth Status								
Richest	-	-	-	-	-	0.040	0.072	0.295
						(0.189)	(0.257)	(0.463)
Richer	-	-	-	-	-	0.084	0.159	0.251
						(0.270)	(0.364)	(0.439)
Middle	-	-	-	-	-	0.124	0.205	0.219
						(0.320)	(0.403)	(0.419)
Poorer	-	-	-	-	-	0.241	0.259	0.154
						(0.415)	(0.437)	(0.366)
Poorest	-	-	-	-	-	0.511	0.305	0.081
						(0.485)	(0.459)	(0.277)
Log (Household Size)	1.639	1.691	1.772	1.802	1.804	1.759	1.725	1.733
	(0.307)	(0.349)	(0.410)	(0.436)	(0.475)	(0.345)	(0.387)	(0.423)
Rural	0.940	0.926	0.869	0.701	0.380	0.864	0.809	0.727
	(0.229)	(0.256)	(0.334)	(0.464)	(0.518)	(0.333)	(0.392)	(0.452)
Religion	0.917	0.924	0.905	0.907	0.913	0.925	0.933	0.897
	(0.267)	(0.258)	(0.290)	(0.295)	(0.301)	(0.255)	(0.250)	(0.308)

Table S2: Sample Characteristics by Wealth and Maternal Education Categories (Nepal)

	Wealth Quintiles					Mother's Education		
	Poorest	Poorer	Middle	Richer	Richest	No Educ.	Primary	Secondary
	Mean (SD)					Mean (SD)		
HAZ	-2.108	-1.882	-1.531	-1.346	-1.101	-1.941	-1.664	-1.248
	(1.490)	(1.349)	(1.272)	(1.234)	(1.277)	(1.369)	(1.293)	(1.367)
WAZ	-1.691	-1.665	-1.441	-1.163	-0.855	-1.714	-1.399	-1.017
	(1.165)	(1.043)	(0.952)	(0.981)	(1.137)	(1.049)	(1.023)	(1.076)
Child Specific Variables								
Child's Age Group								
Age 0-11M	0.171	0.187	0.229	0.190	0.207	0.163	0.194	0.245
	(0.412)	(0.393)	(0.379)	(0.370)	(0.420)	(0.365)	(0.394)	(0.438)
Age 12-35M	0.410	0.399	0.392	0.455	0.353	0.401	0.420	0.397
	(0.538)	(0.493)	(0.441)	(0.470)	(0.495)	(0.484)	(0.492)	(0.499)
Age 36-59M	0.418	0.414	0.379	0.355	0.440	0.436	0.386	0.359
	(0.539)	(0.496)	(0.438)	(0.452)	(0.514)	(0.490)	(0.485)	(0.489)
Male	0.496	0.567	0.444	0.499	0.546	0.492	0.521	0.515
	(0.547)	(0.499)	(0.448)	(0.472)	(0.516)	(0.494)	(0.498)	(0.510)
Birth Size	0.231	0.195	0.161	0.168	0.096	0.206	0.161	0.147
	(0.461)	(0.399)	(0.332)	(0.353)	(0.305)	(0.400)	(0.366)	(0.362)
Birth Order	3.404	2.789	2.270	2.051	1.783	3.313	2.314	1.631
	(2.450)	(1.993)	(1.382)	(1.245)	(1.044)	(2.088)	(1.509)	(0.917)
Mother Specific Variables								
Mother's Age	28.823	26.742	25.568	25.141	27.034	28.525	25.574	24.966
	(7.703)	(6.307)	(4.935)	(4.490)	(4.579)	(6.431)	(5.592)	(4.672)
Maternal Education								
No Education	0.712	0.559	0.506	0.278	0.076	-	-	-
	(0.495)	(0.500)	(0.451)	(0.423)	(0.275)			
Primary	0.188	0.256	0.218	0.211	0.082	-	-	-
	(0.427)	(0.440)	(0.373)	(0.385)	(0.284)			
Secondary	0.101	0.185	0.276	0.512	0.842	-	-	-
	(0.329)	(0.392)	0.403)	(0.472)	(0.379)			
Mother's Height	150.815	150.449	150.908	151.586	152.440	150.483	150.937	152.134
	(5.954)	(5.407)	(4.470)	(4.972)	(5.564)	(5.425)	(4.892)	(5.236)
Mother's BMI	20.473	20.364	20.623	21.281	22.985	20.231	21.214	21.883
	(2.595)	(2.460)	(2.536)	(2.973)	(3.608)	(2.465)	(2.961)	(3.261)
Father Specifc Variables								
Father's Age	33.143	30.471	29.351	30.020	31.984	32.949	29.063	29.451
	(10.207)	(7.680)	(5.296)	(5.383)	(5.339)	(8.062)	(6.640)	(5.553)
Father's Education								
No Education	0.365	0.280	0.230	0.092	0.006	0.410	0.080	0.032
	(0.526)	(0.453)	(0.380)	(0.272)	(0.079)	(0.486)	(0.271)	(0.179)
Primary	0.340	0.310	0.264	0.140	0.083	0.317	0.330	0.095
	(0.518)	(0.466)	(0.398)	(0.328)	(0.286)	(0.459)	(0.469)	(0.299)
Secondary	0.296	0.410	0.506	0.768	0.911	0.273	0.590	0.873
	(0.499)	(0.496)	(0.451)	(0.398)	(0.295)	(0.440)	(0.490)	(0.339)
Hosuehold Specific Variables								
Household Wealth Status								
Richest	-	-	-	-	-	0.023	0.058	0.352
						(0.147)	(0.232)	(0.487)
Richer	-	-	-	-	-	0.102	0.183	0.265
						(0.299)	(0.386)	(0.450)
Middle	-	-	-	-	-	0.253	0.258	0.194
						(0.429)	(0.436)	(0.403)
Poorer	-	-	-	-	-	0.240	0.261	0.112
						(0.422)	(0.438)	(0.322)
Poorest	-	-	-	-	-	0.383	0.240	0.076
						(0.480)	(0.425)	(0.271)
Log (Household Size)	1.769	1.715	1.741	1.754	1.637	1.806	1.671	1.660
	(0.395)	(0.418)	(0.366)	(0.446)	(0.427)	(0.374)	(0.447)	(0.426)
Rural	0.980	0.980	0.961	0.876	0.647	0.954	0.926	0.842
	(0.153)	(0.140)	(0.174)	(0.311)	(0.495)	(0.206)	(0.260)	(0.372)
Religion	0.864	0.804	0.803	0.875	0.855	0.822	0.831	0.866
	(0.374)	(0.400)	(0.359)	(0.312)	(0.365)	(0.378)	(0.373)	(0.347)

Table S3: Age-Specific Maternal Education Gradients in Early Childhood Health (By Educational Categories)

	Panel A			
	Bangladesh (Dependent Variable: HAZ Scores)			
	(Without Father's Education)		(With Father's Education)	
	(1)	(2)	(3)	(4)
	No Education	Primary Education	No Education	Primary Education
Age 0-11 M	-0.072	0.031	-0.035	0.073
	(0.121)	(0.100)	(0.121)	(0.100)
Age 12-35 M	-0.162*	-0.132*	-0.117	-0.089
	(0.091)	(0.070)	(0.090)	(0.069)
Age 36-59 M	-0.132*	-0.121**	-0.087	-0.081
	(0.069)	(0.060)	(0.074)	(0.062)
	N: 7508, R-Sq: 0.207; Mean VIF: 2.83		N: 7505, R-Sq: 0.209; Mean VIF: 2.83	

	Panel B			
	Bangladesh (Dependent Variable: WAZ Scores)			
	(Without Father's Education)		(With Father's Education)	
	(1)	(2)	(3)	(4)
	No Education	Primary Education	No Education	Primary Education
Age 0-11 M	-0.288***	-0.130*	-0.276***	-0.110
	(0.099)	(0.073)	(0.100)	(0.073)
Age 12-35 M	-0.261***	-0.275***	-0.245***	-0.253***
	(0.075)	(0.057)	(0.077)	(0.057)
Age 36-59 M	-0.073	-0.086*	-0.058	-0.068
	(0.060)	(0.050)	(0.063)	(0.052)
	N: 7508, R-Sq: 0.203; Mean VIF: 2.21		N: 7504, R-Sq: 0.204; Mean VIF: 2.22	

	Panel C			
	Nepal (Dependent Variable: HAZ scores)			
	(Without Father's Education)		(With Father's Education)	
	(1)	(2)	(3)	(4)
	No Education	Primary Education	No Education	Primary Education
Age 0-11 M	-0.174	-0.015	-0.151	-0.017
	(0.175)	(0.210)	(0.182)	(0.212)
Age 12-35 M	-0.272**	-0.236	-0.240*	-0.237
	(0.135)	(0.155)	(0.135)	(0.156)
Age 36-59 M	-0.142	-0.000	-0.110	0.002
	(0.118)	(0.133)	(0.125)	(0.136)
	N: 2352, R-Sq: 0.224; Mean VIF: 2.06		N: 2342, R-Sq: 0.221; Mean VIF: 2.11	

	Panel D			
	Nepal (Dependent Variable: WAZ Scores)			
	(Without Father's Education)		(With Father's Education)	
	(1)	(2)	(3)	(4)
	No Education	Primary Education	No Education	Primary Education
Age 0-11 M	-0.326**	-0.131	-0.320**	-0.133
	(0.148)	(0.176)	(0.149)	(0.176)
Age 12-35 M	-0.312***	-0.265**	-0.302***	-0.264**
	(0.112)	(0.130)	(0.113)	(0.129)
Age 36-59 M	-0.278***	-0.076	-0.261**	-0.076
	(0.104)	(0.111)	(0.108)	(0.110)
	N: 2349, R-Sq: 0.189; Mean VIF: 2.07		N: 2339, R-Sq: 0.187; Mean VIF: 2.12	

Note: The numbers in the parenthesis refer to the linearized standard errors that account for survey design and sample weight. *** indicates p< 0.01, ** indicates p < 0.05 and * indicates p <0.1. The table reports regression coefficients of the interaction terms of dummies for each maternal educational category with child age group dummies where secondary education is the reference category. In each panel, we present results from two regression models. Columns (1) and (2) present results from a single regression model that does not control for father's education, and columns (3) and (4) present the results from the same model with father's education as an additional control variable. Each regression model control for the full set of control variables as in Table (4) of the main paper.
